# Host-Directed Virus-Mimicking Particles Interacting with the ACE2 Receptor Competitively Block Coronavirus SARS-CoV-2 Entry

**DOI:** 10.1021/acs.nanolett.3c04430

**Published:** 2024-03-11

**Authors:** Pei Zhang, Erik Niemelä, Sandra López Cerdá, Pasi Sorvisto, Jani Virtanen, Hélder A. Santos

**Affiliations:** † Drug Research Program, Division of Pharmaceutical Chemistry and Technology, Faculty of Pharmacy, University of Helsinki, Helsinki 00014, Finland; ‡ Finncure Oy, Lars Sonckin Kaari 14, Espoo 02600, Finland; § Department of Biomaterials and Biomedical Technology, University Medical Center Groningen, University of Groningen, Ant. Deusinglaan 1, 9713 AV Groningen, The Netherlands

**Keywords:** SARS-CoV-2, Spike S1 receptor binding domain, angiotensin-converting enzyme 2, virus-mimicking particles, microfluidics

## Abstract

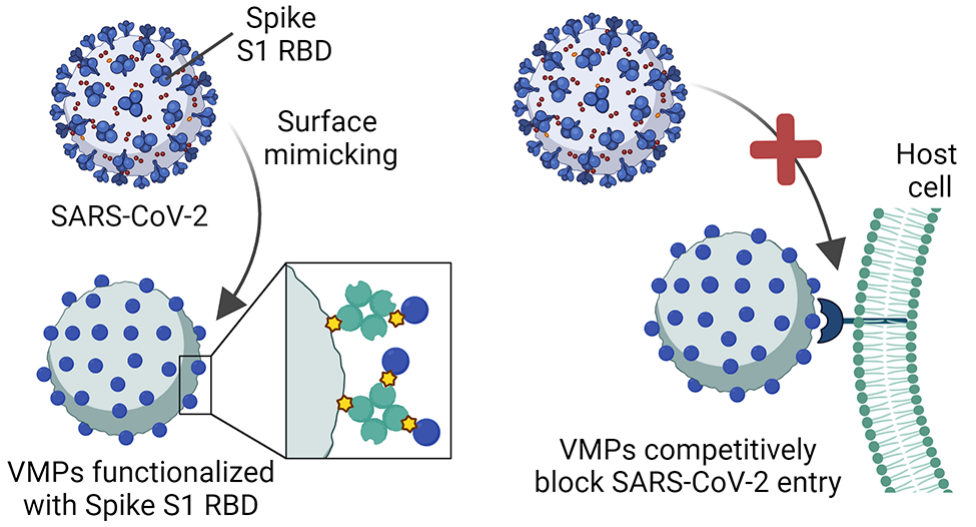

Herein, we fabricate host-directed virus-mimicking particles (VMPs) to block the entry of severe acute respiratory syndrome coronavirus 2 (SARS-CoV-2) into host cells through competitive inhibition enabled by their interactions with the angiotensin-converting enzyme 2 (ACE2) receptor. A microfluidic platform is developed to fabricate a lipid core of the VMPs with a narrow size distribution and a low level of batch-to-batch variation. The resultant solid lipid nanoparticles are decorated with an average of 231 or 444 Spike S1 RBD protrusions mimicking either the original SARS-CoV-2 or its delta variant, respectively. Compared with that of the nonfunctionalized core, the cell uptake of the functionalized VMPs is enhanced with ACE2-expressing cells due to their strong interactions with the ACE2 receptor. The fabricated VMPs efficiently block the entry of SARS-CoV-2 pseudovirions into host cells and suppress viral infection. Overall, this study provides potential strategies for preventing the spread of SARS-CoV-2 or other coronaviruses employing the ACE2 receptor to enter into host cells.

COVID-19, caused by severe acute respiratory syndrome coronavirus 2 (SARS-CoV-2), has swept the globe since 2019, and its reinfections can contribute to additional risk of death, hospitalization, and sequelae regardless of vaccination status.^1^ The attachment of viral particles to host cells is the initial step of SARS-CoV-2 infection, which is mediated by the binding of the receptor binding domain (RBD) within the S1 subunit of the viral Spike glycoprotein to receptors at the host cell surface.^2,3^ The primary receptor for SARS-CoV-2 is angiotensin-converting enzyme 2 (ACE2), a transmembrane enzyme widely expressed in the lung, intestine, liver, heart, vascular endothelium, testis, and kidney.^4^ Impeding the attachment of viral particles to host cells is an efficient way to reduce the level of early infection of SARS-CoV-2. For example, Wang et al. prepared membrane nanoparticles from ACE2-rich cells to act as bait to trap the viral Spike glycoprotein and suppress the entry of SARS-CoV-2 into host cells and, as a result, at least partially blocked SARS-CoV-2 infection *in vitro* and *in vivo.*(5)

Instead of using inhibition or entrapment of the biological activities of viral proteins,^6,7^ an alternative host-directed strategy is conceived through targeting the pathogenic processes in the host cells, such as cellular association, membrane penetration, endosomal escape, virion uncoating, and genome replication, to cease the viral infection.^5,8−10^ For example, the clinically proven camostat mesylate was again identified as an inhibitor of transmembrane protease serine 2 (TMPRSS2), employed by SARS-CoV-2 for Spike protein priming during host cell entry.^11,12^ The host-directed antiviral approach offers advantages in terms of an increased viral resistance threshold, broad-spectrum antiviral action, and improved antiviral outcomes.^13−16^ The host-directed antiviral agents are less likely to lose their capabilities against rapidly evolving and mutating viruses due to the relatively low degree of genetic variability and mutation rate of host cells.^17^

To this end, we developed in this work novel tailorable nanomaterial-based virus-mimicking particles (VMPs) that mimic the pathogen of interest as host-directed agents to block SARS-CoV-2 infection. Specifically, biocompatible solid lipid nanoparticles (SLNs) were functionalized with Spike S1 RBD (Avi-His-Tag, biotin-labeled) protrusions to mimic the structure of SARS-CoV-2 (Scheme 1). Our hypothesis is that the fabricated VMPs could successfully bind to and occupy the ACE2 receptor and, as a result, competitively block the entry of SARS-CoV-2 into host cells and inhibit the early infection.

**Scheme 1:**
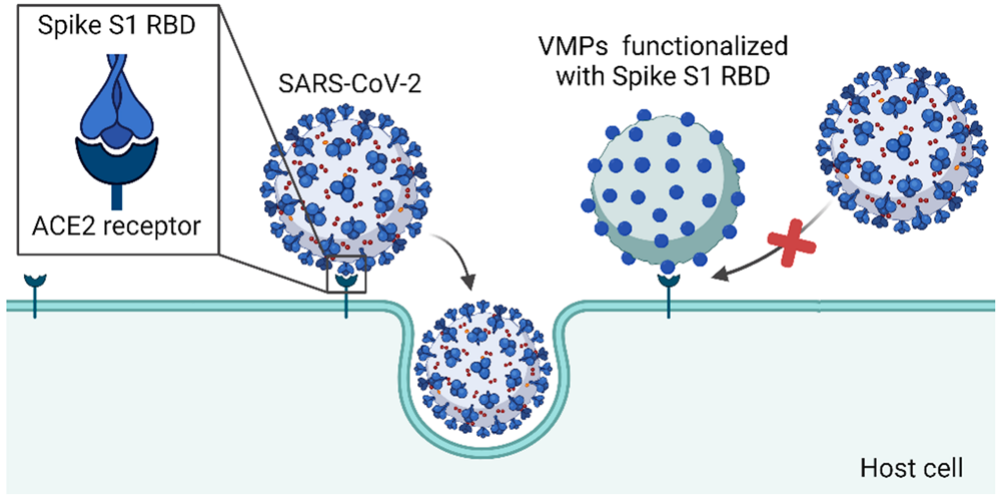
Illustration of the Mechanism of Blocking of SARS-CoV-2 Infection by the Host-Directed VMPs SARS-CoV-2 pathogenesis is initiated by the binding of the Spike S1 RBD to the ACE2 receptor at the host cell surface. The host-directed VMPs can bind to the ACE2 receptor and, as a result, block the entry of the virus into host cells and prevent SARS-CoV-2 infection. This scheme was created with BioRender.com.

## Precise Control of the Lipid Nanoprecipitation Process Enabled by Microfluidics

SARS-CoV-2 is an enveloped and spherical particle with a diameter of ∼120 nm.^18^ To mimic the size and structure of SARS-CoV-2, we fabricated a lipid core composed of 1,2-dioleoyl-3-trimethylammoniumpropane (DOTAP), cholesterol (Chol), dioleoylphosphatidylethanolamine (DOPE), and 1,2-distearoyl-*sn*-glycero-3-phosphoethanolamine-*N*-[biotinyl(polyethylene glycol)-2000] (DSPE-PEG2000-Biotin), with an average size of ∼120 nm. Microfluidics, an advanced technology that can manipulate small (10^–9^ to 10^–18^ L) amounts of fluids at the submillimeter scale, was employed to precisely control the physicochemical properties of the formulation.^19,20^

**Figure 1:**
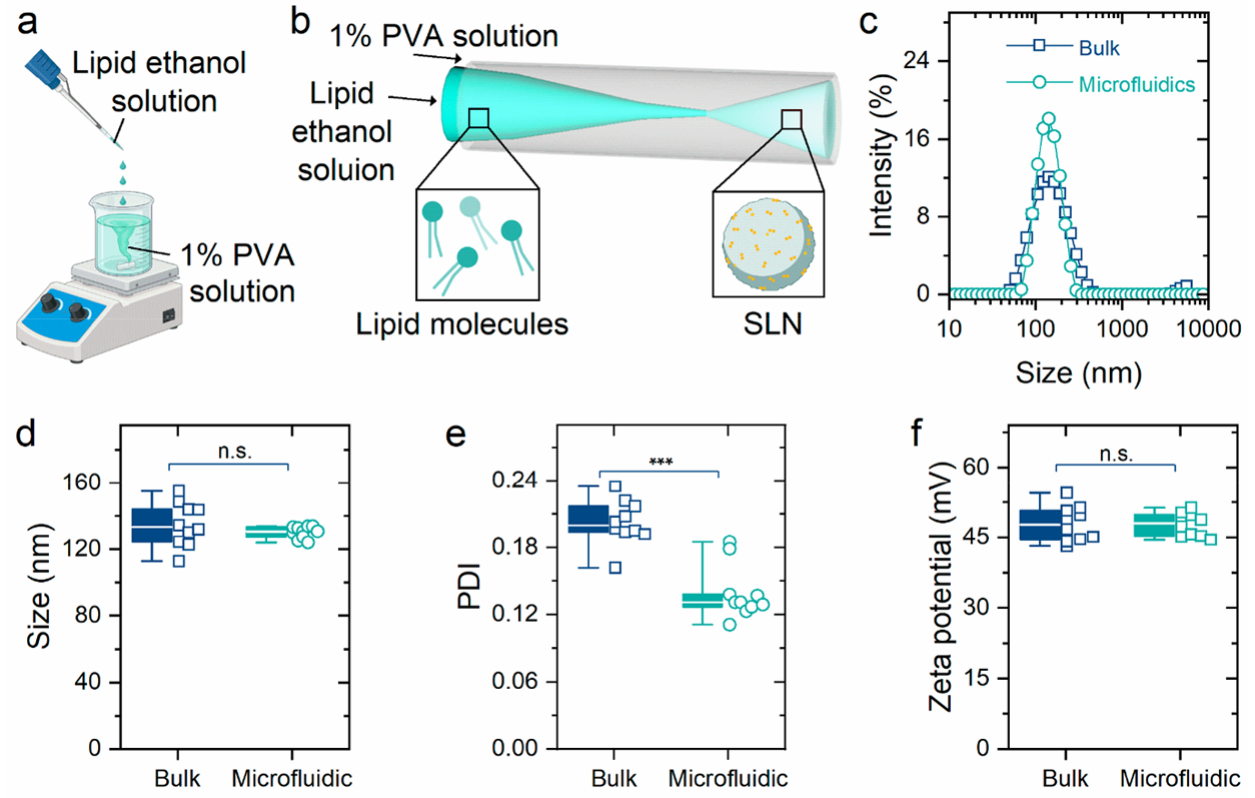
Precise control of the lipid nanoprecipitation process enabled by microfluidics. (a) Schematic illustration of the SLN fabrication process under the bulk condition. This scheme was created with BioRender.com. (b) Schematic illustration of the SLN fabrication process under the microfluidic condition. Part of this scheme (lipid molecules and SLN) was created with BioRender.com. (c) Intensity–size distribution curves of SLN prepared by bulk and microfluidic methods. (d) Average particle sizes, (e) PDIs, and (f) ζ potentials of SLN prepared by bulk and microfluidic methods in different batches (Student’s *t* test; *n* = 10; microfluidic group vs the corresponding bulk group; ***P < 0.001; n.s., not significant). The box plots indicate the minimum value, first quartile, median, third quartile, and maximum value.

In this study, the SLN was fabricated by the nanoprecipitation method under conventional bulk and microfluidic conditions.^21^ For the bulk nanoprecipitation process, the lipid ethanol solution was added dropwise to an aqueous solution of poly(vinyl alcohol) (PVA, 1%, w/v) that was being continuously stirred at 300 rpm (Figure 1a). For the microfluidic nanoprecipitation process, the lipid solution and PVA aqueous solution were pumped into the inner capillary and the space between the inner and outer capillary of a co-flow microfluidic device, respectively (Figure 1b). The lipid molecules self-assembled into SLN due to the diffusion of water into the ethanol phase. Although the hydrodynamic size of SLN prepared by the bulk method was close to that obtained with the microfluidic process, the deficient control of the mixing process and unstable mass transfer for bulk condition resulted in a higher polydispersity and partial agglomeration with an average size of 4955 nm (Figure 1c). By contrast, SLN fabricated by the microfluidic method revealed a monomodal and narrower size distribution, which could be attributed to precise fluid control and rapid microscale mixing in the microfluidic device.

To illustrate the precise control capability of the microfluidic method with respect to the nanoprecipitation process, 10 batches of SLN prepared by bulk and microfluidic processes were characterized. The average hydrodynamic size of SLN fabricated through the bulk method varied from 112.9 to 155.1 nm, while that of SLN fabricated through the microfluidic method varied in the range of 124.1–133.8 nm (Figure 1d). The polydispersity index (PDI) of SLN fabricated by the microfluidic method (0.139 ± 0.024) was significantly (*P* < 0.001) lower than that of the bulk method (0.202 ± 0.020), indicating a more homogeneous size distribution caused by the better mixing performance in the microscale device (Figure 1e). Furthermore, the SLN prepared by the microfluidic process displayed higher batch-to-batch reproducibility in terms of ζ potential (44.5–51.3 mV) compared with the bulk method (43.2–54.5 mV) (Figure 1f). Therefore, the developed microfluidic platform could improve the controllability and reproducibility for the SLN engineering process.

## Functionalization of SLN with the Spike S1 RBD Programmed by Streptavidin–Biotin Interaction

**Figure 2:**
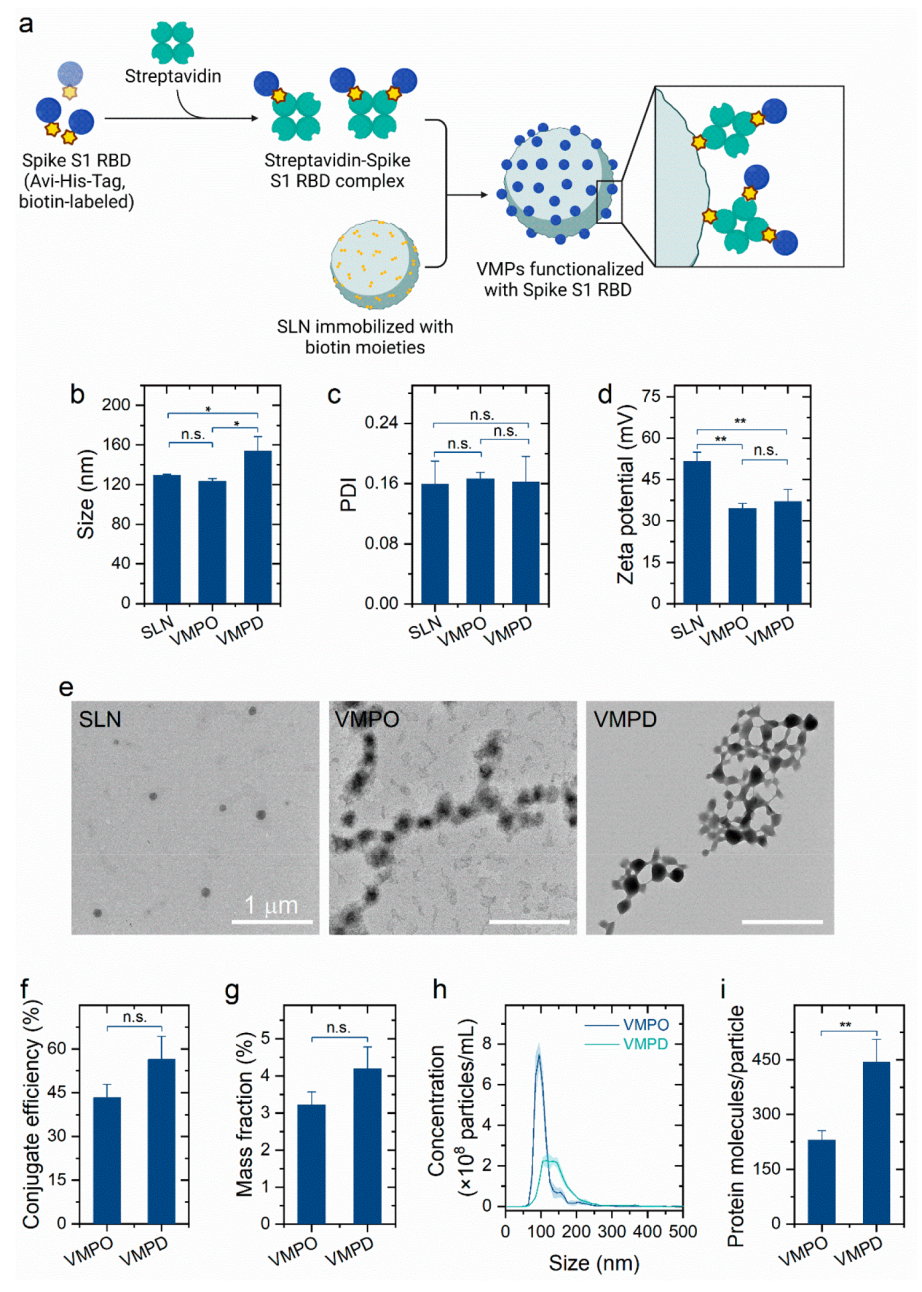
Functionalization of SLN with the Spike S1 RBD programmed by streptavidin–biotin interaction. (a) Schematic illustration of the functionalization of SLN with the Spike S1 RBD through streptavidin–biotin interaction. This scheme was created with BioRender.com. Influence of the functionalization with the Spike S1 RBD on (b) particle size, (c) PDI, and (d) ζ potential [one-way analysis of variance (ANOVA) with post hoc Bonferroni’s test; *n* = 3; *P < 0.05; **P < 0.01; n.s., not significant]. (e) Transmission electron microscope images of SLN, VMPO, and VMPD. (f) Conjugation efficiency and (g) mass fraction of the Spike S1 RBD in VMPO and VMPD (Student’s *t* test; *n* = 3; VMPD group vs the corresponding VMPO; n.s., not significant). (h) Representative histogram showing the particle size distribution vs nanoparticle concentration of VMPO and VMPD obtained by nanoparticle tracking analysis. (i) Number of Spike S1 RBD molecules carried on the surface of VMPO and VMPD (Student’s *t* test; *n* = 3; VMPD group vs the corresponding VMPO; **P < 0.01).

For DSPE-PEG2000-Biotin, the biotin moieties tend to be located on the particle surface when self-assembling because of the hydrophobicity of DSPE and hydrophilicity of PEG.^22^ To mimic the structure of SARS-CoV-2, the surface of SLN immobilized with biotin moieties was functionalized with Spike S1 RBD [Avi-His-Tag, biotin-labeled (Figure S1)] protrusions derived from the original SARS-CoV-2 (VMPO) and its delta variant (VMPD) through a streptavidin linker (Figure 2a). Streptavidin is a tetrameric protein that has a high affinity for biotin (*K*_a_ = 10^13^–10^15^ M^–1^).^23^ It has four biotin binding sites symmetrically located in the exterior region, which potentially lead to multiple captures of biotinylated particles on one tetravalent streptavidin and, as a result, cause particle aggregation.^23^ To avoid particle aggregation, the Spike S1 RBD (Avi-His-Tag, biotin-labeled) was initially mixed with streptavidin in a 1.5:1 mole ratio to form a streptavidin–Spike S1 RBD complex. This complex was then bound to the biotin moieties on the SLN surface, and the hindrance resistance caused by the bound Spike S1 RBD is supposed to prevent multiple captures of particles on one streptavidin and the resultant aggregation.

The average size of the SLN was 129.7 nm with a PDI of 0.159 ± 0.031, as measured by dynamic light scattering (Figure 2b,c). As expected, there was no sign of particle aggregation after functionalization, indicated by the uniform peak of the hydrodynamic radius at 123.4 ± 2.9 nm for VMPO and 154.3 ± 14.2 nm for VMPD (Figure 2b and Figure S2). The size of VMPs was homogeneous with PDIs (0.167 ± 0.008 for VMPO and 0.162 ± 0.034 for VMPD) that were comparable to that of SLN (Figure 2c). The SLN was positively charged with a ζ potential of 51.7 mV, which decreased (*P* < 0.01) to ∼34.6 mV for VMPO and ∼37.0 mV for VMPD after functionalization (Figure 2d). The decrease in the ζ potential demonstrated the successful conjugation of the streptavidin–Spike S1 RBD complex to the SLN. The SLN and VMPs were regarded as stable colloidal suspension systems because of their high ζ potential (>30 mV) and the resultant strong repulsion between nanoparticles.^24^ As a result, the nanoparticles showed excellent colloidal stability at 4 °C for at least 200 days (Figure S3). After functionalization, the exterior surface of SLN was buried with a gray layer (Figure 2e), which might be composed of the streptavidin–Spike S1 RBD complex and indicate the successful conjugation of the Spike S1 RBD to the surface of VMPs. The slight discrepancy in the particle size was observed through dynamic light scattering and transmission electron microscopy, because dynamic light scattering measures the hydrodynamic diameter of the nanoparticle, including the solvation layer, whereas transmission electron microscopy presents an estimation of the projected area diameter in a dry state.

The efficient functionalization of VMPs with the Spike S1 RBD was measured with a Micro BCA. The conjugate efficiency of the Spike S1 RBD, defined as the weight percentage of conjugated molecules among the added molecules, was 43.3% for VMPO and 56.4% for VMPD (Figure 2f). This corresponded to mass fractions, the amount of the Spike S1 RBD conjugated per unit weight of VMPs, of 3.22% for VMPO and 4.20% for VMPD (Figure 2g). The size distribution measured by nanoparticle tracking analysis showed a mean particle radii of 109.4 nm for VMPO and 145.9 nm for VMPD with 90% of the particles being <145.4 nm for VMPO and <199.1 nm for VMPD, confirming the narrow size distribution of the nanoparticles (Figure 2h). The total nanoparticle concentration was represented by the area under the curve, which equaled 3 × 10^9^ particles/mL for VMPO and 2.03 × 10^9^ particles/mL for VMPD. Accordingly, the number of Spike S1 RBD molecules carried on each VMP was calculated by considering the mass of the conjugated Spike S1 RBD and the particle concentration of VMPs, which corresponded to an average of 231 Spike S1 RBDs for VMPO (Figure 2i). By contrast, a VMPD carried ∼444 Spike S1 RBDs on the particle surface, which was significantly (*P* < 0.01) higher than that of VMPO. Benefiting from the remarkable affinity between streptavidin and biotin, the number of Spike S1 RBD molecules bound to each VMP was obviously higher than that reported previously, which might potentially improve the blocking efficiency of the VMPs.^25,26^

## Enhanced Cell Uptake of VMPs Mediated by the Interaction with the ACE2 Receptor

The Spike protein of SARS-CoV-2 was reported to interact with human cerebrovascular cells, including endothelial cells, pericytes, and smooth muscle cells, mediated by ACE2.^27^ We hypothesized that the prepared VMPs functionalized with the Spike S1 RBD bind to ACE2 on the cell surface, which may enhance their uptake into the host cells. To prove that the fabricated VMPs bind to ACE2, we evaluated the cell uptake of VMPs with a human lung carcinoma A549 cell line expressing ACE2 (A549-ACE2), human non-small cell lung cancer Calu-3 cells, and human colorectal adenocarcinoma Caco-2 cells, which are expressed with ACE2 and highly susceptible to SARS-CoV-2 infection.^28−30^ By contrast, A549 cells, which express a negligible level of ACE2 and, thus, are weakly susceptible to SARS-CoV-2 infection, served as the control.^31^

**Figure 3:**
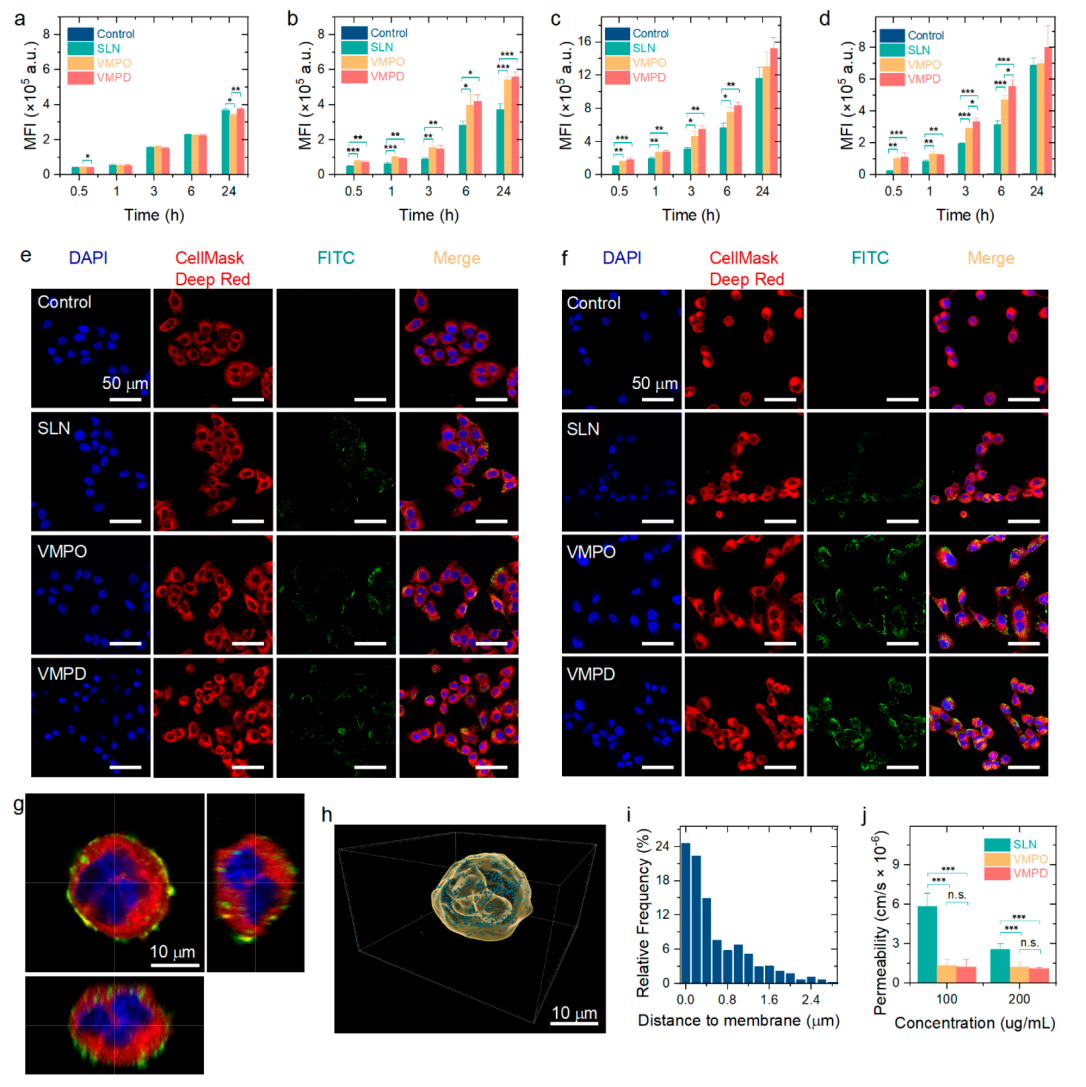
Enhanced cell uptake of VMPs mediated by the interaction with the ACE2 receptor. Cell uptake of FITC-labeled SLN, VMPO, and VMPD by (a) A549, (b) A549-ACE2, (c) Calu-3, and (d) Caco-2 cells after different incubation times (0.5, 1, 3, 6, and 24 h) quantified by flow cytometry (one-way ANOVA with post hoc Bonferroni’s test; *n* = 3; *P < 0.05; **P < 0.01; ***P < 0.001). Laser scanning confocal microscope images revealing the cell uptake of FITC-labeled SLN, VMPO, and VMPD (20 μg/mL) by (e) A549 and (f) A549-ACE2 cells after treatment for 3 h. (g) Orthogonal view of the laser scanning confocal microscope image revealing the subcellular localization of FITC-labeled VMPO (20 μg/mL) in Calu-3 cells after treatment for 3 h. (h) Three-dimensional surface and dot reconstructions revealing the internalization of FITC-labeled VMPO (20 μg/mL) in Calu-3 cells after treatment for 3 h. The wheat surface identifies the cytomembrane stained with CellMask Deep Red, while the steel blue surface represents FITC-labeled VMPO. (i) Distance distribution of FITC-labeled VMPO from the Calu-3 cell membrane. (j) Apparent permeability coefficients of SLN, VMPO, and VMPD (100 and 200 μg/mL) after incubation at 37 °C for 18 h (one-way ANOVA with post hoc Bonferroni’s test; *n* = 4; ***P < 0.001; n.s., not significant).

Before the cell uptake of SLN and VMPs was evaluated, a cell viability assay was performed to test the biocompatibility of the nanoparticles. The viability study suggested that the SLN and VMPs were nontoxic up to 200 μg/mL and, thus, can be potentially used to block SARS-CoV-2 infection (Figure S4). To prove the interactions between VMPs and ACE2, the uptake of SLN and VMPs was evaluated quantitatively through flow cytometry. Fluorescein isothiocyanate (FITC)-labeled DSPE-PEG was employed for the synthesis of fluorescent SLN. The FITC-labeled SLN and VMPs were taken up rapidly with >40% of the cells exhibiting nanoparticle fluorescence after 0.5 h, which may be attributed to their positive surface charge (Figure S5). The mean fluorescence intensity (MFI) increased with incubation time from 0.5 to 24 h, indicating the continual cell uptake of SLN and VMPs (Figure 3a–d). For A549 cells with negligible levels of ACE2, there was no significant difference between the uptake levels for the SLN and VMP groups (Figure 3a). For ACE2-expressing cells (A549-ACE2, Calu-3, and Caco-2), the functionalization of VMPs with Spike S1 RBD protrusions significantly enhanced their cellular uptake compared to that of the bare SLN after incubation for ≤6 h (Figure 3b-d). This enhanced effect disappeared for Calu-3 and Caco-2 cells as the incubation time increased to 24 h, possibly due to the exhaustion of VMPs.

The enhanced cell uptake of VMPs after functionalization with Spike S1 RBD protrusions was further confirmed by visualizing their cellular distribution through a laser scanning confocal microscope (Figure 3e,f and Figures S6 and S7). To indicate the distribution of FITC-labeled nanoparticles (green), the cell nucleus was stained with 4′,6-diamidino-2-phenylindole (DAPI, blue), while the cytomembrane was stained with CellMask Deep Red. Consistent with the results of flow cytometry, the fluorescence intensity of the FITC-labeled VMPs was stronger than that of the FITC-labeled SLN group for A549-ACE2, Calu-3, and Caco-2 cells, while a negligible difference was observed for A549 cells. Representative *z*-stack images (Figure 3g and Figure S8) demonstrated that part of the VMPO was internalized into Calu-3 cells after incubation for 3 h, while the others adsorbed onto the cytomembrane because of their interaction with cells. Three-dimensional models of the cytomembrane (wheat) and nanoparticles (steel blue) were generated to visualize the subcellular localization of FITC-labeled nanoparticles (Figure 3h). Approximately 53% of VMPO was distributed within the Calu-3 cells with a distance to the cymembrane of ≥0.5 μm (Figure 3i), indicating the successful cellular internalization of VMPO.

To indicate nanoparticle permeability across the pulmonary epithelial epithelium, the diffuse ability of SLN and VMPs is evaluated employing a parallel artificial membrane permeability assay (PAMPA) kit (Figure 3j). The apparent permeability coefficient of SLN was 5.8 × 10^–6^ cm/s for 100 μg/mL and 2.6 × 10^–6^ cm/s for 200 μg/mL, demonstrating its good permeability.^32^ The coefficients decreased significantly (*P* < 0.001) after functionalization with Spike S1 RBD protrusions derived from the original SARS-CoV-2 (1.3 × 10^–6^ cm/s for 100 μg/mL and 1.2 × 10^–6^ cm/s for 200 μg/mL) and its delta variant (1.2 × 10^–6^ cm/s for 100 μg/mL and 1.1 × 10^–6^ cm/s for 200 μg/mL). The significant decrease in permeability ability can be attributed to the functionalization with the Spike S1 RBD, which covered the hydrophobic lipid surface with hydrophilic proteins.

In summary, the fabricated SLN and VMPs exhibited excellent cell compatibility. The conjugated Spike S1 RBD protrusions enhanced the cell uptake of VMPs, confirming the interaction between VMPs and ACE2 on the cytomembrane. The exterior surface of VMPs buried by the Spike S1 RBD ensured their significantly decreased permeability, which may lead to good compatibility, weaker side effects, and potential clinical application of VMPs.

## VMPs Efficiently Blocked SARS-CoV-2 Infection through Interaction with ACE2

**Figure 4:**
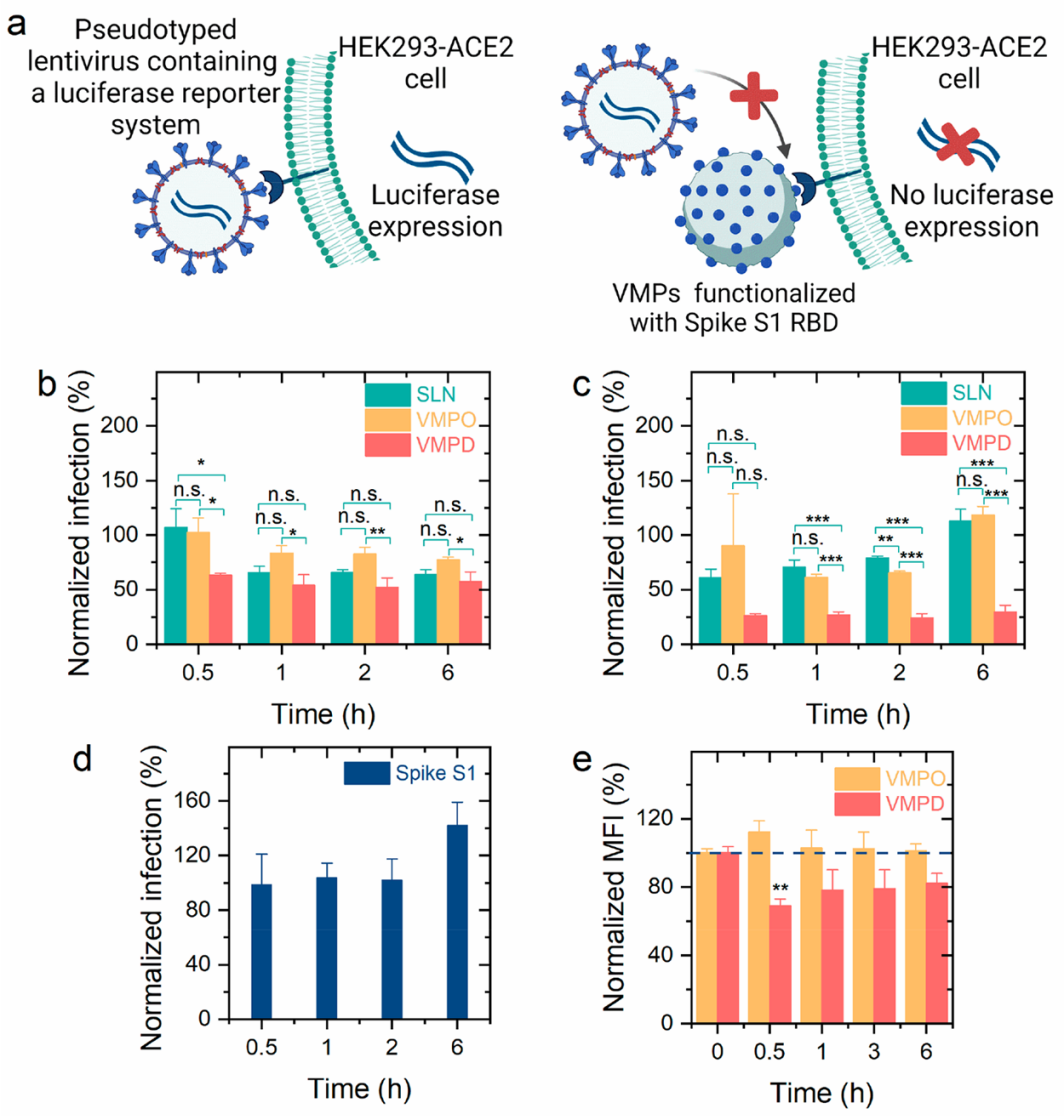
VMPs efficiently blocked SARS-CoV-2 infection through interaction with ACE2. (a) Schematic illustration of the pseudoviral infection assay. This scheme was created with BioRender.com. Effects of nanoparticles (SLN and VMPs) at concentrations of (b) 20 and (c) 100 μg/mL and (d) the Spike S1 RBD (1 μg/μL, Avi-His-tag, biotin-labeled) on blocking SARS-CoV-2 pseudoviral infection (one-way ANOVA with posthoc Bonferroni’s test; *n* = 3; *P < 0.05; **P < 0.01; ***P < 0.001; n.s., not significant). (e) Effect of the Spike S1 RBD (Avi-His-Tag, 1 μg/μL) on the cell uptake of FITC-labeled VMPO and VMPD by A549-ACE2 cells after incubation for 1 h (one-way ANOVA with posthoc Bonferroni’s test; *n* = 3; the 0.5, 1, 3, and 6 h groups were compared with the corresponding 0 h group; **P < 0.01).

We hypothesized that the VMPs functionalized with Spike S1 RBD protrusions could attach to and occupy ACE2, provide steric hindrance to inhibit viral attachment, and, as a result, block entry of the virus into host cells and prevent SARS-CoV-2 infection. A pseudoviral infection assay, which was developed to evaluate neutralizing antibodies against SARS-CoV-2, was modified to study the effect of VMPs on blocking SARS-CoV-2 infection (Figure 4a).^33^ Briefly, after being pretreated with nanoparticles (SLN and VMPs, 20 and 100 μg/mL) and the Spike S1 RBD (1 μg/μL, Avi-His-tag, biotin-labeled), human embryonic kidney 293 cells expressing ACE2 (HEK293-ACE2) were infected with Spike (SARS-CoV-2) pseudotyped lentivirus containing a luciferase reporter system. The pseudoviral infection was measured by employing a One-Step luciferase assay. The fewer pseudotyped lentiviruses that entered the cells, the lower the intensity of the emitted light. This pseudoviral infection assay is quantitative and sensitive and can be carried out in biosafety level 2 facilities.^34^

In the pseudoviral infection assay, a neutralizing antibody was served as the negative control (Figure S9). Treatment with SLN and VMPs suppressed SARS-CoV-2 pseudovirions infection, while the free Spike S1 protein did not indicate any inhibitory capacity (Figure 4b–d). As shown by the cell uptake study, SLNs attached to and are internalized into cells rapidly because of their positive surface charge. The attachment of SLNs repelled the pseudovirions from cells, and therefore, SLN could weakly inhibit (normalized infection as low as ∼60%) pseudoviral infection. When the nanoparticle concentration was 20 μg/mL, the inhibitory capacity of VMPD was significantly (*P* < 0.05) higher than that of SLN with a 0.5 h nanoparticle pretreatment; as the pretreatment time increased, negligible difference was observed. For a nanoparticle concentration of 100 μg/mL, the VMPD maintained a strong inhibitory capacity (normalized infection varied in the range of 24–30%) for 6 h and significantly (*P* < 0.001) decreased the level of pseudoviral infection compared with both SLN and VMPO. The more efficient blockade provided by VMPD could be ascribed to the higher binding affinity of the delta Spike S1 RBD for ACE2 as well as the larger amount of the Spike S1 RBD conjugated to each VMPD surface.^35^

To further study the interaction between VMPs and the ACE2 receptor, we investigated the effect of the Spike S1 RBD (Avi-His-Tag, 1 μg/μL) on the uptake of VMPs by A549-ACE2 cells (Figure 4e). The cells were pretreated with the Spike S1 RBD (Avi-His-Tag, 1 μg/μL) for different periods of time (0, 0.5, 1, 3, and 6 h), followed by incubation with FITC-labeled VMPs for 1 h. The Spike S1 RBD greatly (*P* < 0.01) reduced the level of cell uptake of VMPD after pretreatment for 0.5 h, indicating ACE2, as the receptor of the Spike S1 RBD, was involved in the internalization of VMPD; the inhibitory effect was barely observed as the pretreatment time increased, which may be attributed to the fast exhaustion of the Spike S1 RBD. Surprisingly, the Spike S1 RBD had a negligible influence on the cell uptake of VMPO. This result demonstrated that VMPO might efficiently replace the Spike S1 RBD bond to ACE2 receptor and, as a result, could potentially serve as a blocker not only before but also during SARS-CoV-2 infection.

In summary, we have fabricated two types of host-directed VMPs against SARS-CoV-2 infection enabled by blocking entry of the virus into the host cells through the ACE2 receptor. The lipid core of the VMPs was manufactured employing a microfluidic platform. This platform was amenable to sufficient control during the precipitation process, and as a result, the fabricated SLN revealed a narrow size distribution and high reproducibility. SLN was functionalized with abundant Spike S1 RBD protrusions derived from the original SARS-CoV-2 and its delta variant because of the extraordinarily high affinity of the biotin–streptavidin interaction. Specifically, the resultant VMPD carried a significantly larger amount of Spike S1 RBD protrusions on its surface. The interaction between VMPs and the ACE2 receptor was proven by their enhanced cell uptake into ACE2-expressing cells. Because of the interaction, VMPs efficiently blocked SARS-CoV-2 pseudoviral infection. In particular, VMPD effectively maintained the normalized pseudoviral infection to 24–30% for ≤6 h at a dosage of 100 μg/mL. These results highlight the potential of VMPs as candidates against SARS-CoV-2 infection, shedding light on future strategies for combating global COVID-19 pandemics. Furthermore, the host-directed VMPs may also provide protection against other coronaviruses employing the ACE2 receptor for entry, such as HCoV-NL63 and SARS-CoV, and potentially offer insights for pathogenic outbreak control both locally and globally.^36^

## Supporting Information Available

Additional figures of the amino acid sequences of the Spike S1 RBD (Avi-His-Tag, biotin-labeled), size, stability, cytotoxicity, cell update (flow cytometry analysis and laser scanning confocal microscope imaging), and pseudoviral infection (PDF)

## References

[ref1] Bowe B.; Xie Y.; Al-Aly Z. Acute and postacute sequelae associated with SARS-CoV-2 reinfection. Nat. Med. 2022, 28 (11), 2398–2405. 10.1038/s41591-022-02051-3. 36357676 PMC9671810

[ref2] Jackson C. B.; Farzan M.; Chen B.; Choe H. Mechanisms of SARS-CoV-2 entry into cells. Nat. Rev. Mol. Cell Biol. 2022, 23 (1), 3–20. 10.1038/s41580-021-00418-x. 34611326 PMC8491763

[ref3] Tai W.; He L.; Zhang X.; Pu J.; Voronin D.; Jiang S.; Zhou Y.; Du L. Characterization of the receptor-binding domain (RBD) of 2019 novel coronavirus: implication for development of RBD protein as a viral attachment inhibitor and vaccine. Cell. Mol. Immunol. 2020, 17 (6), 613–620. 10.1038/s41423-020-0400-4. 32203189 PMC7091888

[ref4] Masre S. F.; Jufri N. F.; Ibrahim F. W.; Abdul Raub S. H. Classical and alternative receptors for SARS-CoV-2 therapeutic strategy. Rev. Med. Virol. 2021, 31 (5), 1 10.1002/rmv.2207. PMC7883063 33368788

[ref5] Wang C.; Wang S.; Chen Y.; Zhao J.; Han S.; Zhao G.; Kang J.; Liu Y.; Wang L.; Wang X.; et al. Membrane nanoparticles derived from ACE2-rich cells block SARS-CoV-2 infection. ACS Nano 2021, 15 (4), 6340–6351. 10.1021/acsnano.0c06836. 33734675

[ref6] Nieva J. L.; Madan V.; Carrasco L. Viroporins: structure and biological functions. Nat. Rev. Microbiol. 2012, 10 (8), 563–574. 10.1038/nrmicro2820. 22751485 PMC7097105

[ref7] Nakagawa K.; Makino S. Mechanisms of coronavirus nsp1-mediated control of host and viral gene expression. Cells 2021, 10 (2), 300 10.3390/cells10020300. 33540583 PMC7912902

[ref8] Schwegmann A.; Brombacher F. Host-directed drug targeting of factors hijacked by pathogens. Sci. Signaling 2008, 1 (29), re8 10.1126/scisignal.129re8. 18648074

[ref9] Law G. L.; Korth M. J.; Benecke A. G.; Katze M. G. Systems virology: host-directed approaches to viral pathogenesis and drug targeting. Nat. Rev. Microbiol. 2013, 11 (7), 455–466. 10.1038/nrmicro3036. 23728212 PMC4028060

[ref10] Robinson P. C.; Liew D. F. L.; Tanner H. L.; Grainger J. R.; Dwek R. A.; Reisler R. B.; Steinman L.; Feldmann M.; Ho L. P.; Hussell T.; et al. COVID-19 therapeutics: Challenges and directions for the future. Proc. Natl. Acad. Sci. U. S. A. 2022, 119 (15), e2119893119 10.1073/pnas.2119893119. 35385354 PMC9169797

[ref11] Hoffmann M.; Kleine-Weber H.; Schroeder S.; Krüger N.; Herrler T.; Erichsen S.; Schiergens T. S.; Herrler G.; Wu N. H.; Nitsche A.; et al. SARS-CoV-2 cell entry depends on ACE2 and TMPRSS2 and is blocked by a clinically proven protease inhibitor. Cell 2020, 181 (2), 271–280.e8. 10.1016/j.cell.2020.02.052. 32142651 PMC7102627

[ref12] Gordon D. E.; Jang G. M.; Bouhaddou M.; Xu J.; Obernier K.; White K. M.; O’Meara M. J.; Rezelj V. V.; Guo J. Z.; Swaney D. L.; et al. A SARS-CoV-2 protein interaction map reveals targets for drug repurposing. Nature 2020, 583 (7816), 459–468. 10.1038/s41586-020-2286-9. 32353859 PMC7431030

[ref13] Kaufmann S. H.; Dorhoi A.; Hotchkiss R. S.; Bartenschlager R. Host-directed therapies for bacterial and viral infections. Nat. Rev. Drug Discovery 2018, 17 (1), 35–56. 10.1038/nrd.2017.162. 28935918 PMC7097079

[ref14] Beigel J. H.; Nam H. H.; Adams P. L.; Krafft A.; Ince W. L.; El-Kamary S. S.; Sims A. C. Advances in respiratory virus therapeutics – A meeting report from the 6th isirv Antiviral Group conference. Antiviral Res. 2019, 167, 45–67. 10.1016/j.antiviral.2019.04.006. 30974127 PMC7132446

[ref15] Trimarco J. D.; Nelson S. L.; Chaparian R. R.; Wells A. I.; Murray N. B.; Azadi P.; Coyne C. B.; Heaton N. S. Cellular glycan modification by B3GAT1 broadly restricts influenza virus infection. Nat. Commun. 2022, 13 (1), 6456 10.1038/s41467-022-34111-0. 36309510 PMC9617049

[ref16] Wei J.; Patil A.; Collings C. K.; Alfajaro M. M.; Liang Y.; Cai W. L.; Strine M. S.; Filler R. B.; DeWeirdt P. C.; Hanna R. E.; et al. Pharmacological disruption of mSWI/SNF complex activity restricts SARS-CoV-2 infection. Nat. Genet. 2023, 55 (3), 471–483. 10.1038/s41588-023-01307-z. 36894709 PMC10011139

[ref17] Kumar N.; Sharma S.; Kumar R.; Tripathi B. N.; Barua S.; Ly H.; Rouse B. T. Host-directed antiviral therapy. Clin. Microbiol. Rev. 2020, 33 (3), e00168-19 10.1128/CMR.00168-19. 32404434 PMC7227448

[ref18] Liu Y. C.; Kuo R. L.; Shih S. R. COVID-19: The first documented coronavirus pandemic in history. Biomed. J. 2020, 43 (4), 328–333. 10.1016/j.bj.2020.04.007. 32387617 PMC7199674

[ref19] Sackmann E. K.; Fulton A. L.; Beebe D. J. The present and future role of microfluidics in biomedical research. Nature 2014, 507 (7491), 181–189. 10.1038/nature13118. 24622198

[ref20] Whitesides G. M. The origins and the future of microfluidics. Nature 2006, 442 (7101), 368–373. 10.1038/nature05058. 16871203

[ref21] Zhang P.; Liu Y.; Feng G.; Li C.; Zhou J.; Du C.; Bai Y.; Hu S.; Huang T.; Wang G.; et al. Controlled interfacial polymer self-assembly coordinates ultrahigh drug loading and zero-order release in particles prepared under continuous flow. Adv. Mater. 2023, 35 (22), 2211254 10.1002/adma.202211254. 36802103

[ref22] Zhai J.; Hinton T. M.; Waddington L. J.; Fong C.; Tran N.; Mulet X.; Drummond C. J.; Muir B. W. Lipid–PEG conjugates sterically stabilize and reduce the toxicity of phytantriol-based lyotropic liquid crystalline nanoparticles. Langmuir 2015, 31 (39), 10871–10880. 10.1021/acs.langmuir.5b02797. 26362479

[ref23] Xia N.; Shumaker-Parry J. S.; Zareie M. H.; Campbell C. T.; Castner D. G. A streptavidin linker layer that functions after drying. Langmuir 2004, 20 (9), 3710–3716. 10.1021/la035864n. 15875404

[ref24] Das P.; Das M. K. Chapter 4 - Production and physicochemical characterization of nanocosmeceuticals. In Nanocosmeceuticals; Das M. K., Ed.; Academic Press, 2022; pp 95–138.

[ref25] Tan T. K.; Rijal P.; Rahikainen R.; Keeble A. H.; Schimanski L.; Hussain S.; Harvey R.; Hayes J. W. P.; Edwards J. C.; McLean R. K.; et al. A COVID-19 vaccine candidate using SpyCatcher multimerization of the SARS-CoV-2 spike protein receptor-binding domain induces potent neutralising antibody responses. Nat. Commun. 2021, 12 (1), 542 10.1038/s41467-020-20654-7. 33483491 PMC7822889

[ref26] Zha L.; Chang X.; Zhao H.; Mohsen M. O.; Hong L.; Zhou Y.; Chen H.; Liu X.; Zhang J.; Li D.; et al. Development of a vaccine against SARS-CoV-2 based on the receptor-binding domain displayed on virus-like particles. Vaccines (Basel) 2021, 9, 395 10.3390/vaccines9040395. 33923573 PMC8073353

[ref27] McQuaid C.; Solorzano A.; Dickerson I.; Deane R. Uptake of severe acute respiratory syndrome coronavirus 2 spike protein mediated by angiotensin converting enzyme 2 and ganglioside in human cerebrovascular cells. Front. Neurosci. 2023, 17, 1117845 10.3389/fnins.2023.1117845. 36875642 PMC9980911

[ref28] Chang C. W.; Parsi K. M.; Somasundaran M.; Vanderleeden E.; Liu P.; Cruz J.; Cousineau A.; Finberg R. W.; Kurt-Jones E. A. A newly engineered A549 cell line expressing ACE2 and TMPRSS2 is highly permissive to SARS-CoV-2, including the Delta and Omicron variants. Viruses 2022, 14 (7), 1369 10.3390/v14071369. 35891350 PMC9318744

[ref29] Tseng C.-T. K.; Tseng J.; Perrone L.; Worthy M.; Popov V.; Peters C. J. Apical entry and release of severe acute respiratory syndrome-associated coronavirus in polarized Calu-3 lung epithelial cells. J. Virol. 2005, 79 (15), 9470–9479. 10.1128/JVI.79.15.9470-9479.2005. 16014910 PMC1181546

[ref30] Rouaud F.; Méan I.; Citi S. The ACE2 receptor for coronavirus entry is localized at apical cell—cell junctions of epithelial cells. Cells 2022, 11 (4), 627 10.3390/cells11040627. 35203278 PMC8870730

[ref31] Ma D.; Chen C. B.; Jhanji V.; Xu C.; Yuan X. L.; Liang J. J.; Huang Y.; Cen L. P.; Ng T. K. Expression of SARS-CoV-2 receptor ACE2 and TMPRSS2 in human primary conjunctival and pterygium cell lines and in mouse cornea. Eye 2020, 34 (7), 1212–1219. 10.1038/s41433-020-0939-4. 32382146 PMC7205026

[ref32] Đanić M.; Pavlović N.; Stanimirov B.; Lazarević S.; Vukmirović S.; Al-Salami H.; Mikov M. PAMPA model of gliclazide permeability: The impact of probiotic bacteria and bile acids. Eur. J. Pharm. Sci. 2021, 158, 105668 10.1016/j.ejps.2020.105668. 33301903

[ref33] Lei C.; Qian K.; Li T.; Zhang S.; Fu W.; Ding M.; Hu S. Neutralization of SARS-CoV-2 spike pseudotyped virus by recombinant ACE2-Ig. Nat. Commun. 2020, 11 (1), 2070 10.1038/s41467-020-16048-4. 32332765 PMC7265355

[ref34] Nie J.; Li Q.; Wu J.; Zhao C.; Hao H.; Liu H.; Zhang L.; Nie L.; Qin H.; Wang M.; et al. Quantification of SARS-CoV-2 neutralizing antibody by a pseudotyped virus-based assay. Nat. Protoc. 2020, 15 (11), 3699–3715. 10.1038/s41596-020-0394-5. 32978602

[ref35] Abeywardhana S.; Premathilaka M.; Bandaranayake U.; Perera D.; Peiris L. D. C. In silico study of SARS-CoV-2 spike protein RBD and human ACE-2 affinity dynamics across variants and Omicron subvariants. J. Med. Virol. 2023, 95 (1), e28406 10.1002/jmv.28406. 36519577 PMC9877981

[ref36] Lan J.; Chen P.; Liu W.; Ren W.; Zhang L.; Ding Q.; Zhang Q.; Wang X.; Ge J. Structural insights into the binding of SARS-CoV-2, SARS-CoV, and hCoV-NL63 spike receptor-binding domain to horse ACE2. Structure 2022, 30 (10), 1432–1442.e4. 10.1016/j.str.2022.07.005. 35917815 PMC9341007

