# Supplementary material for: Host-Directed Virus-Mimicking Particles Interacting with the ACE2 Receptor Competitively Block Coronavirus SARS-CoV-2 Entry
Source: Nano Lett. 2024 Mar 11;24(14):4064–71. doi: 10.1021/acs.nanolett.3c04430 (PMC11010226; doi:10.1021/acs.nanolett.3c04430)
Supplement: Supplementary file 1 — nl3c04430_si_001.pdf [file nl3c04430_si_001.pdf]

Host-directed virus-mimicking particles interacting with the ACE2 receptor competitively block coronavirus SARS-CoV-2 entry

*Pei Zhang[1,2], Erik Niemelä[2], Sandra López Cerdá[1], Pasi Sorvisto[2], Jani Virtanen[2], Hélder A. Santos[1,3]**

[1]Drug Research Program, Division of Pharmaceutical Chemistry and Technology, Faculty of Pharmacy, University of Helsinki, Helsinki 00014, Finland

[2]Finncure Oy, Lars Sonckin Kaari 14, Espoo 02600, Finland

[3]Department of Biomaterials and Biomedical Technology, University Medical Center Groningen, University of Groningen, Ant. Deusinglaan 1, 9713 AV Groningen, The Netherlands

Email: h.a.santos@umcg.nl

EXPERIMENTAL METHODS

Fabrication of the microfluidic device. The microfluidic device was fabricated through coaxially aligned assembling of two borosilicate glass capillaries (World Precision Instruments, USA).[1-3] The inner capillary with an inner diameter (i.d.) of 580 μm and outer diameter (o.d.) of 1000 μm was tapered using a micropipette puller (P-97, Sutter Instrument, USA). The fine tip was polished, and the diameter of the orifice was further enlarged to approximately 100 μm. This inner capillary was coaxially inserted into the outer cylindrical capillary with i.d. of 1120 μm and o.d. of 1500 μm. The capillaries were fixed on a glass slide and sealed as required employing a transparent epoxy resin.

Preparation of SLN through microfluidic precipitation method. The SLN composed of DOTAP (Avanti Polar Lipids, Inc., USA), Chol (≥99%; Merck Limited, USA), DOPE (Avanti Polar Lipids, Inc., USA), and DSPE-PEG2000-Biotin (Avanti Polar Lipids, Inc., USA) was prepared through nanoprecipitation method employing a microfluidic device. The inner phase, ethanol solution of DOTAP (4.84 mg/mL), Chol (0.54 mg/mL), DOPE (4 mg/mL), and DSPE-PEG-Biotin (0.62 mg/mL), was pumped into the inner capillary. The outer phase, an aqueous solution of PVA (1%, w/v), flowed through the space between the inner and outer capillary in the same direction. The lipid molecules precipitated into nanoparticles as the diffusion of water into the ethanol phase. The flow rate of the inner and outer phases was 1mL/min, controlled by PHD 2000 pumps (Harvard Apparatus, USA). To thoroughly remove the ethanol and PVA, the fabricated SLN were washed with Milli-Q water employing Nanosep® Centrifugal Device with Omega™ Membrane 100K (Pall Corporation, USA) for 6 times. The FITC-labelled SLN was prepared with modified method, replacing the inner phase with an ethanol solution of DOTAP (4.22 mg/mL), Chol (0.54 mg/mL), DOPE (4 mg/mL), DSPE-PEG-Biotin (0.62 mg/mL) and distearoyl phosphoethanolamine-polyethylene glycol-fluorescein isothiocyanate (DSPE-PEG-FITC, 0.62 mg/mL, Nanosoft Polymers, USA). The flow velocity of the inner and outer phase

was 1 and 0.82 mL/min, respectively. The following purification process was as same as that of SLN.

Preparation of SLN through bulk precipitation method. To fabricate SLN by bulk precipitation method, ethanol solution of DOTAP (4.84 mg/mL), Chol (0.54 mg/mL), DOPE (4 mg/mL), and DSPE-PEG-Biotin (0.62 mg/mL, 0.5 mL) was added dropwise into an aqueous solution of PVA (1%, w/v, 0.5 mL) under stirring at 300 rpm. The precipitated nanoparticles were purified by rinsing with Milli-Q water using Nanosep® Centrifugal Device with Omega™ Membrane 100K (Pall Corporation, USA) for 6 times.

Streptavidin-biotin-programmed functionalization of SLN with Spike S1 RBD. The SLN was functionalized with Spike S1 RBD derived from the original and delta variant SARS-CoV-2 through biotin-streptavidin interaction to form VMPO and VMPD, respectively. Specifically, Spike S1 RBD (Avi-His-Tag, biotin-labeled, BPS Bioscience, USA) solution (90 µg/mL, 0.5 mL) was added to streptavidin (Merck Limited, USA) solution (120 µg/mL, 0.5 mL) dropwise. After stirring at 4 °C for 2 h, SLN suspension (1 mg/mL, 0.5 mL) was added into the mixture, which was further stirred at 4 °C for 3 h. To remove the unconjugated Spike S1 RBD, the mixture was dialyzed against Milli-Q water employing Spectra/Por® dialysis membrane with molecular weight cut-off of 1000 kDa (Biotech CE tubing, Spectrum Labs, USA) for 48 h. For the functionalization of SARS-CoV-2 Spike S1 RBD (B.1.617.2, delta variant, Avi-His-Tag, biotin-labeled, BPS Bioscience, USA), the stirring time was decreased to 0.5 and 2 h, respectively. The FITC-labelled VMPO and VMPD were prepared with the same method.

Characterization of SLN and VMPs. The size and size distribution of SLN and VMPs were analyzed through dynamic light scattering using Zetasizer Nano ZS (Malvern Instruments Ltd., UK). The analysis was performed with a disposable polystyrene cuvette (Sarstedt AG & Co., Germany) at an angle of 173° at 25 °C. Their zeta-potential was measured employing Zetasizer

Nano ZS equipped with a disposable folder capillary cell (DTS1070, Malvern Instruments Ltd., UK). Samples were dispersed in Milli-Q water during measurement. Morphological characteristics of the fabricated SLN and VMPs stained with ammonium molybdate (1%) were observed by JEOL JEM-1400 transmission electron microscopy (JEOL Ltd., Japan). The mass of conjugated Spike S1 RBD in VMPs was measured with Micro BCA™ Protein Assay Kit (Thermo Fisher Scientific, USA) following the manufacturer's protocol. Mixtures of Spike S1 RBD and streptavidin (mole ratio 1.5:1) with different concentrations served as the standards. Conjugate efficiency, the weight percentage of conjugated Spike S1 RBD among the added Spike S1 RBD, was calculated as [(weight of conjugated Spike S1 RBD/weight of added Spike S1 RBD) × 100%]. Mass fraction, the amount of Spike S1 RBD conjugated to per unit weight of VMPs, was calculated as [(weight of conjugated Spike S1 RBD/weight of VMPs) × 100%]. For the stability study, SLN and VMPs were stored at 4 °C for 100 and 200 days. Afterward, their size and size distribution were measured through dynamic light scattering using Zetasizer Nano ZS (Malvern Instruments Ltd., UK).

The particle concentration of VMPs was measured through nanoparticle tracking analysis with Nanosight LM14C (Malvern Panalytical Ltd., UK). The VMPs were captured and analyzed with the built-in NanoSight Software NTA version 3.4.4 (Malvern Panalytical Ltd., UK). The camera level was fixed at 15 to ensure all VMPs were visible without signal saturation. The detection threshold was set as 5 to include most of the observed VMPs while excluding indistinct ones. VMP suspensions were diluted with Milli-Q water to concentrations of 0.5-1 μg/mL and injected into the sample chamber by a sterile syringe (HENKE-JECT®, Henke Sass Wolf GmbH, Germany) until reaching the nozzle tip. For each measurement, five consecutive videos were recorded for 30 s with a 405 nm laser (blue) and scientific CMOS camera. Based on the measured mass of conjugated Spike S1 RBD and particle concentration of VMPs, the number of conjugated Spike S1 RBD per VMP was calculated by the following Equation (1):

$$N = \frac{mN_A}{CM_{mol}} \qquad (1)$$

where, N is the number of conjugated Spike S1 RBD per VMP, m is the mass of conjugated Spike S1 RBD, N_A is Avogadro constant=6.02×10^{23}, C is particle concentration of VMPs, and M_{mol} is the molar mass of Spike S1 RBD.

Cell culture. The *in vitro* cytotoxicity and uptake study were conducted with human lung carcinoma A549 cells, A549-ACE2 cells, human non-small-cell lung cancer Calu-3 cells, and human colorectal adenocarcinoma Caco-2 cells. All cells were cultured in Dulbecco's modified Eagle medium (DMEM) with 4.5 g/L glucose (Life Technologies Gibco, USA). For A549 and Caco-2 cells, the DMEM medium was supplemented with heat inactivated fetal bovine serum (10%, Life Technologies Gibco, USA), l-glutamine (1%, GE Healthcare Lifesciences, USA), non-essential amino acids (1%, GE Healthcare Lifesciences, USA), penicillin (100 IU/mL, GE Healthcare Lifesciences, USA), and streptomycin (100 μg/mL, GE Healthcare Lifesciences, USA). Three more antibiotics, normocin (100 μg/mL, InvivoGen, USA), puromycin (0.5 μg/mL, InvivoGen, USA), and hygromycin B gold (300 μg/mL, Invivogen, USA), were further added into the DMEM medium for A549-ACE2 cells. Calu-3 cells were cultured in the DMEM medium supplemented with heat inactivated fetal bovine serum (20%), l-glutamine (1%), penicillin (100 IU/mL), and streptomycin (100 μg/mL). The HEK-293T-ACE2 cells for *in vitro* pseudoviral infection assay were cultured in Growth Medium 1N (BPS Bioscience, USA) and plated in Thaw Medium 1 (BPS Bioscience, USA). All cells were maintained at 37 °C under a 5% CO_2 atmosphere and 95% relative humidity. The cell culture medium was changed every other day.

In vitro cytotoxicity study. To perform the *in vitro* cytotoxicity study, A549, A549-ACE2, Calu-3, and Caco-2 cells were seeded on a 96-well plate (Corning Incorporated, USA) at a density of 2×10^4 cells per well, followed by overnight incubation for attachment. Afterward,

the culture medium was changed into the fresh medium with SLN and VMPs at different concentrations (10, 20, 50, 100, 200, and 500 μg/mL). Cell culture medium without or with cells served as the blank and positive control, respectively. After incubation for 24, 48, and 72 h, the cell viability was measured using CellTiter-Glo luminescent assay (Promega Corporation, USA) with Varioskan LUX multimode microplate reader (Thermo Fisher Scientific, USA).

In vitro cell uptake study. The *in vitro* cell uptake of SLN and VMPs was evaluated through flow cytometry analysis and confocal imaging. For flow cytometry analysis, A549, A549-ACE2, Calu-3, and Caco-2 cells were seeded in 12-well plates (Corning Incorporated, USA) at a density of 3×10^5 cells per well. The cells were incubated overnight for attachment, followed by treatment with FITC-labelled SLN, VMPO, and VMPD (20 μg/mL) for 0.5, 1, 3, 6, and 24 h. Afterward, the cells were washed with phosphate buffered saline (PBS) twice and detached by trypsin (0.25%, v/v, GE Healthcare Lifesciences, USA). The detached cells were washed with PBS twice and analyzed on a BD LSR-II Cell Analyzer flow cytometer (Becton Dickinson, USA). The fluorescence on the cell surface was quenched by incubating with trypan blue (0.005%, Thermo Fisher Scientific Inc, USA) for 15 min. The cells were washed with PBS and analyzed again by BD LSR-II Cell Analyzer flow cytometer.

For confocal imaging, A549, A549-ACE2, Calu-3, and Caco-2 cells were seeded in Lab-Tek chambered borosilicate coverglass (8-well, Thermo Fisher Scientific, USA) at a density of 2.5×10^4 cells per well. After incubating overnight for attachment, the cells were cocultured with FITC-labelled SLN, VMPO, and VMPD (20 μg/mL) for 3 h. Cells treated with culture medium without any particles served as control. After washing with PBS twice, the cytomembrane was stained with CellMask Deep Red (5 μg/mL in PBS, 200 μL, Invitrogen, USA), while the cell nucleus was stained with DAPI (2.48 μg/mL, 200 μL, Thermo Fisher Scientific Inc, USA). Finally, the cells were imaged by LEICA SP8 STED confocal microscope

(Leica Microsystems, Germany). The 3D image visualization and reconstruction were performed through Imaris (Oxford Instruments, UK).

Permeability assay. The permeability of SLN and VMPs was evaluated using PAMPA Kit (BioAssay Systems, USA), with a 96-well filter plate as the permeation donor compartment and a 96-well receiver plate as the acceptor. Lecithin dodecane solution (4%, 5 μL) was added to the membrane of each well. Immediately after the application of the artificial membrane, the donor plates were added with test nanoparticle suspensions (100 and 200 μg/mL, 200 μL), while the acceptor plates were filled with PBS (300 μL). All analyzes were performed in quadruplicate. The nanoparticle-filled donor plates were carefully installed into the acceptor plate wells, ensuring the contact of the underside of the membranes with the acceptor solution without air bubbles. After incubating under constant shaking (150 rpm) for 18 h at 37 °C, samples were withdrawn from the acceptor plates. Nanoparticle suspensions diluted to 40 and 80 μg/mL served as equilibrium standards, and PBS was employed as blank control. The absorbance spectrum (200-500 nm) for acceptor solutions, equilibrium standards, and blank controls were read through a UV-1600PC UV-Vis Spectrophotometer (VWR International Oy, Finland) in 10 nm intervals to determine the peak absorbance of test nanoparticles. The apparent permeability coefficient of the nanoparticles was calculated by the following Equation (2):

$$P_{app} = C \times -\ln\left(1 - \frac{OD_A - OD_B}{OD_E - O\ \ _B}\right)\ cm/s \qquad (2)$$

where, P_{app} is the apparent permeability coefficient, OD_A, OD_E, and OD_B are the peak absorbance of acceptor solutions, equilibrium standards, and blank controls, respectively, and C is a constant, 7.72×10^{-6}.

In vitro pseudoviral infection assay. Pseudoviral infection assay was conducted to evaluate the effect of VMPs on blocking the viral entry into host cells and preventing SARS-CoV-2

infection.[4] Briefly, HEK-293T-ACE2 cells were seeded into a 96-well plate at a density of 5×10^3 cells per well and incubated overnight for attachment. The medium was replaced with fresh medium with Spike S1 RBD (1 μg/μL, Avi-His-tag, biotin-labeled, BPS Bioscience, USA), SLN (20 and 100 μg/mL) and VMPs (20 and 100 μg/mL). After incubating for 0.5, 1, 2, or 6 h, Spike (SARS-CoV-2) pseudotyped lentivirus (luciferase reporter, 5 μL, BPS Bioscience, USA) was added to each well. Bald lentiviral pseudovirion (luciferase reporter, BPS Bioscience, USA) and Spike S1 neutralizing antibody (SARS-CoV-2, Clone:414-1, BPS Bioscience, USA) were employed as negative controls. The neutralizing antibody (10 μL, final concentration of 100 nM) was preincubated with the pseudotyped lentivirus for 30 min before adding to the cells. Cells treated with only pseudotyped lentivirus served as the positive control. At 24 h post-infection, the pseudotyped lentivirus was removed and the cells were overlaid with fresh medium. Following additional 24 h incubation, the pseudoviral infection was measured employing One-Step luciferase assay (BPS Bioscience, USA) with POLARstar Omega microplate reader (BMG Labtech, Germany). The experiments were conducted in triplicate, and pseudoviral infection was normalized with the positive control group.

Cell uptake inhibition assay. The effect of Spike S1 RBD (Avi-His-Tag, BPS Bioscience, USA) on the cell uptake of FITC-labelled VMPs was evaluated on A549-ACE2 cells through flow cytometry analysis. Briefly, A549-ACE2 cells were seeded in 12-well plates (Corning Incorporated, USA) at a density of 3×10^5 cells per well. After overnight incubation, the cells were pretreated with Spike S1 RBD (Avi-His-Tag, 1 μg/μL) for 0.5, 1, 3, and 6 h, followed by the coincubation with FITC-labelled VMPs (20 μg/mL) for 1 h. The cells were detached by trypsin (0.25%, v/v, GE Healthcare Lifesciences, USA) and washed with PBS twice. The cell uptake was analyzed through a BD LSR-II Cell Analyzer flow cytometer. After analysis, the fluorescence on the cell surface was quenched by trypan blue (0.005%, Thermo Fisher

Scientific Inc, USA). The quenched cells were washed with PBS and analyzed again by BD LSR-II Cell Analyzer flow cytometer.

Statistical analysis. Results are expressed as mean ± standard deviation (s.d.) for at least three independent experiments. Statistical analyses were performed (Origin 2021b, Origin Lab Corporation, USA) as indicated in the figure legends. The levels of significance were set at probabilities of $^{*}P < 0.05$, $^{**}P < 0.01$, and $^{***}P < 0.001$.

SUPPORTING FIGURES

a

RVQPTESIVRFPNITNLCPFGEVFNATRFASVYAWNR
KRISNCVADYSVLYNSASFSTFKCYGVSPTKLNDLCF
TNVYADSFVIRGDEVRQIAPGQTGKIADYNYKLPDDF
TGCVIAWNSNNLDSKVGGNYNYLYRLFRKSNLKPFE
RDISTEIYQAGSTPCNGVEGFNCYFPLQSYGFQPTN
GVGYQPYRVVVLSFELLHAPATVCGPKKSTNLVKNK
CVNFEFGGGLNDIFEAQKIEWHEGGGHHHHHH

Spike S1 RBD
Linker
Avi
His

b

QPTESIVRFPNITNLCPFGEVFNATRFASVYAWNRKR
ISNCVADYSVLYNSASFSTFKCYGVSPTKLNDLCFTN
VYADSFVIRGDEVRQIAPGQTGKIADYNYKLPDDFTG
CVIAWNSNNLDSKVGGNYNYRYRLFRKSNLKPFERD
ISTEIYQAGSKPCNGVEGFNCYFPLQSYGFQPTNGV
GYQPYRVVVLSFELLHAPATVCGPKKSTNLVKNKCV
NFNFEFGGGLNDIFEAQKIEWHEGGGHHHHHH

Spike S1 RBD
Linker and Avi
Linker and His

Figure S1. The amino acid sequence of the Spike S1 RBD (Avi-His-Tag, biotin-labeled) derived from original (a) and delta variant (b) SARS-CoV-2.

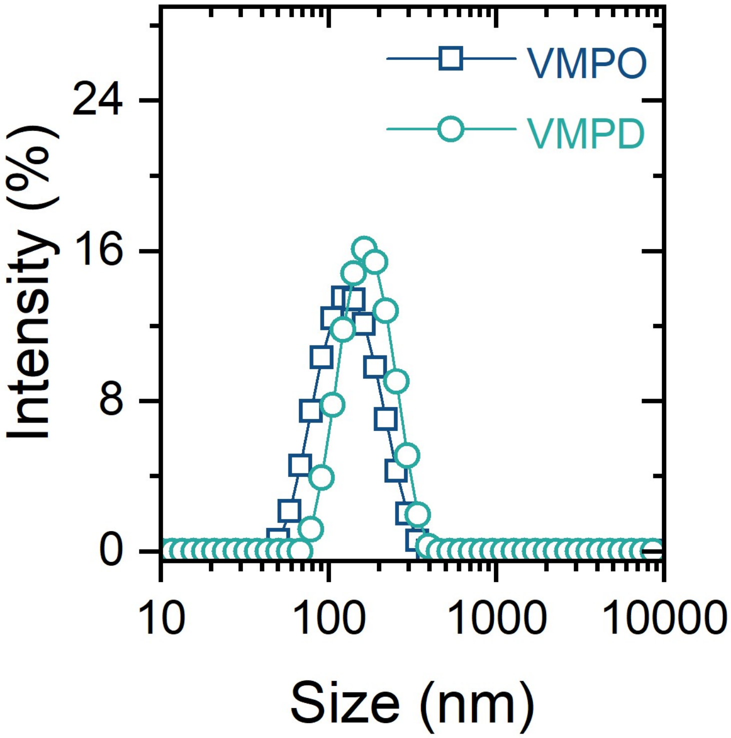

Figure S2. The intensity size distribution curves of VMPO and VMPD.

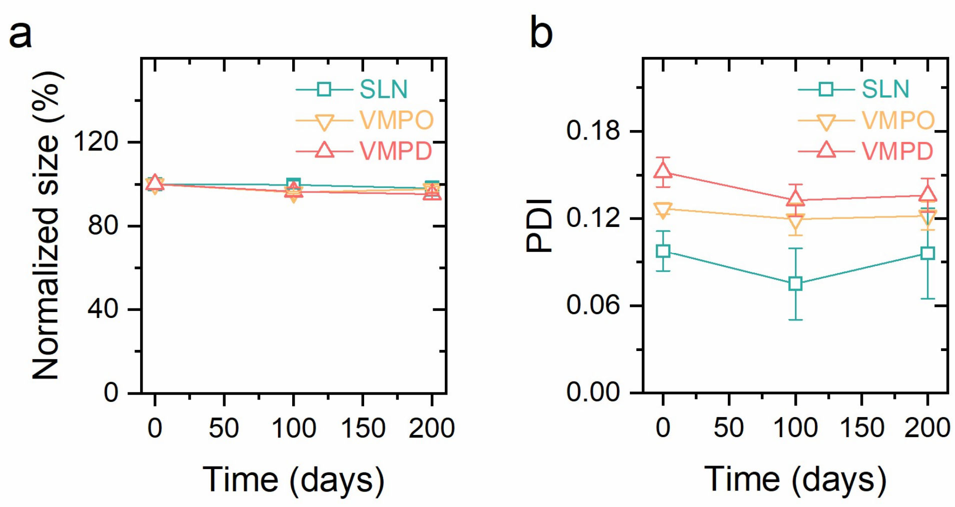

Figure S3. The normalized size (a) and PDI (b) of SLN, VMPO and VMPD storing at 4 °C.

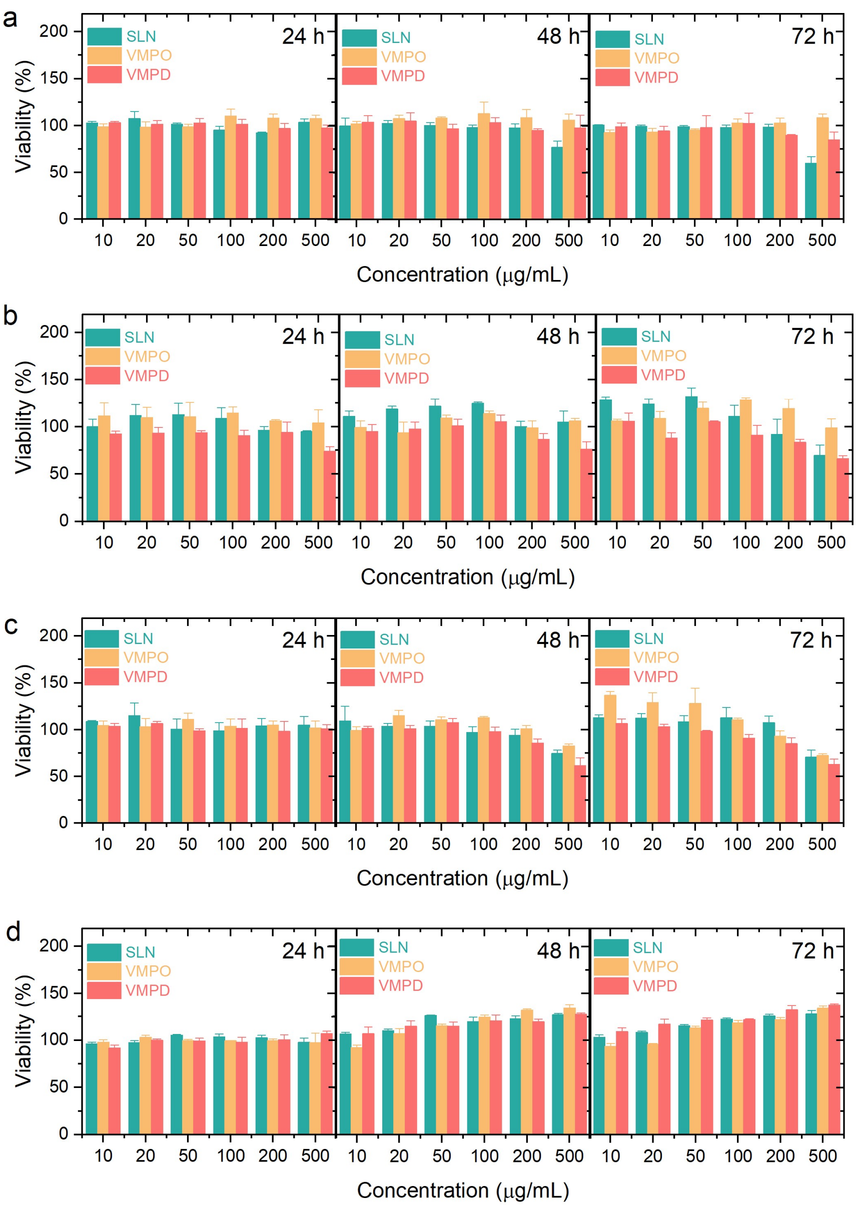

Figure S4. Cell compatibility of SLN and VMPs. (a-d) Cytotoxicity of SLN, VMPO, and VMPD on A549 (a), A549-ACE2 (b), Calu-3 (c), and Caco-2 cells (d) at 37 °C after 24, 48, and 72 h incubation at different concentrations (10, 20, 50, 100, 200, and 500 μg/mL). The viability was measured by CellTiter-Glo® luminescence assay.

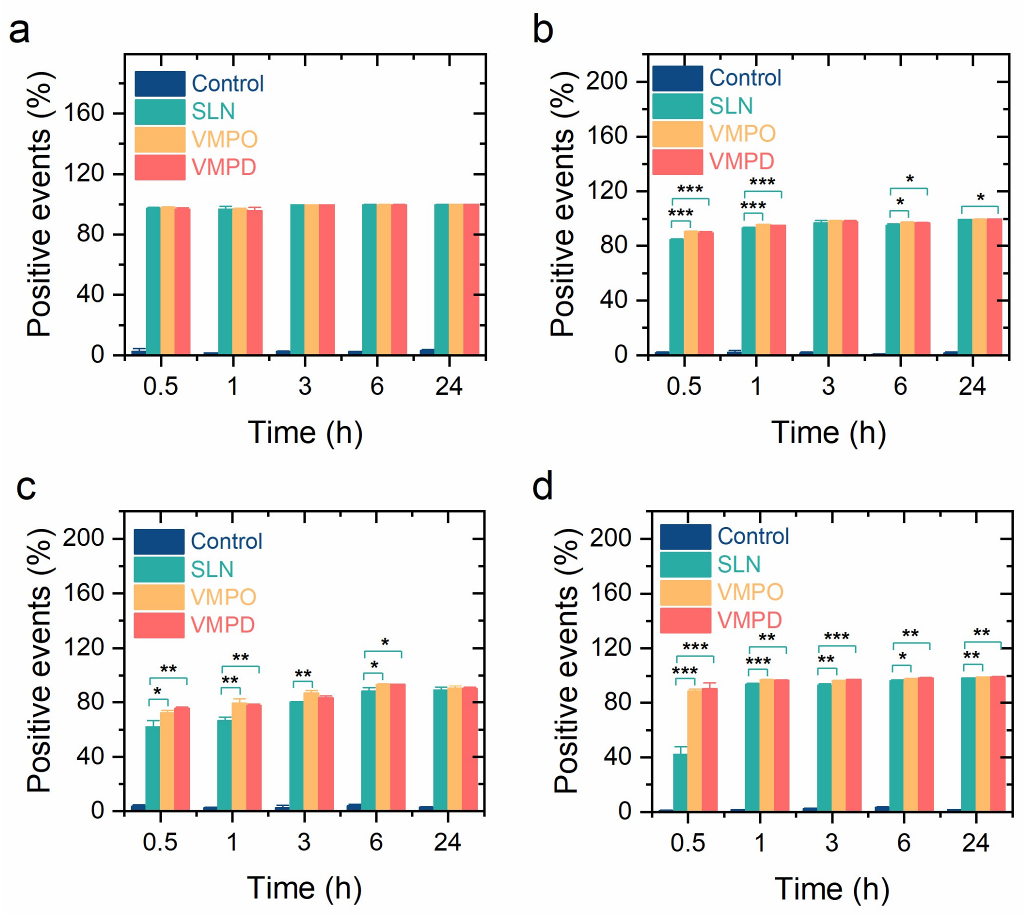

Figure S5. Positive events of A549 (a), A549-ACE2 (b), Calu-3 (c) and Caco-2 (d) cells treated with FITC-labelled SLN, VMPO and VMPD for different time (0.5, 1, 3, 6 and 24 h) quantified by flow cytometry (one-way ANOVA with post-hoc Bonferroni's test; n=3; *$P < 0.05$, **$P < 0.01$ and ***$P < 0.001$).

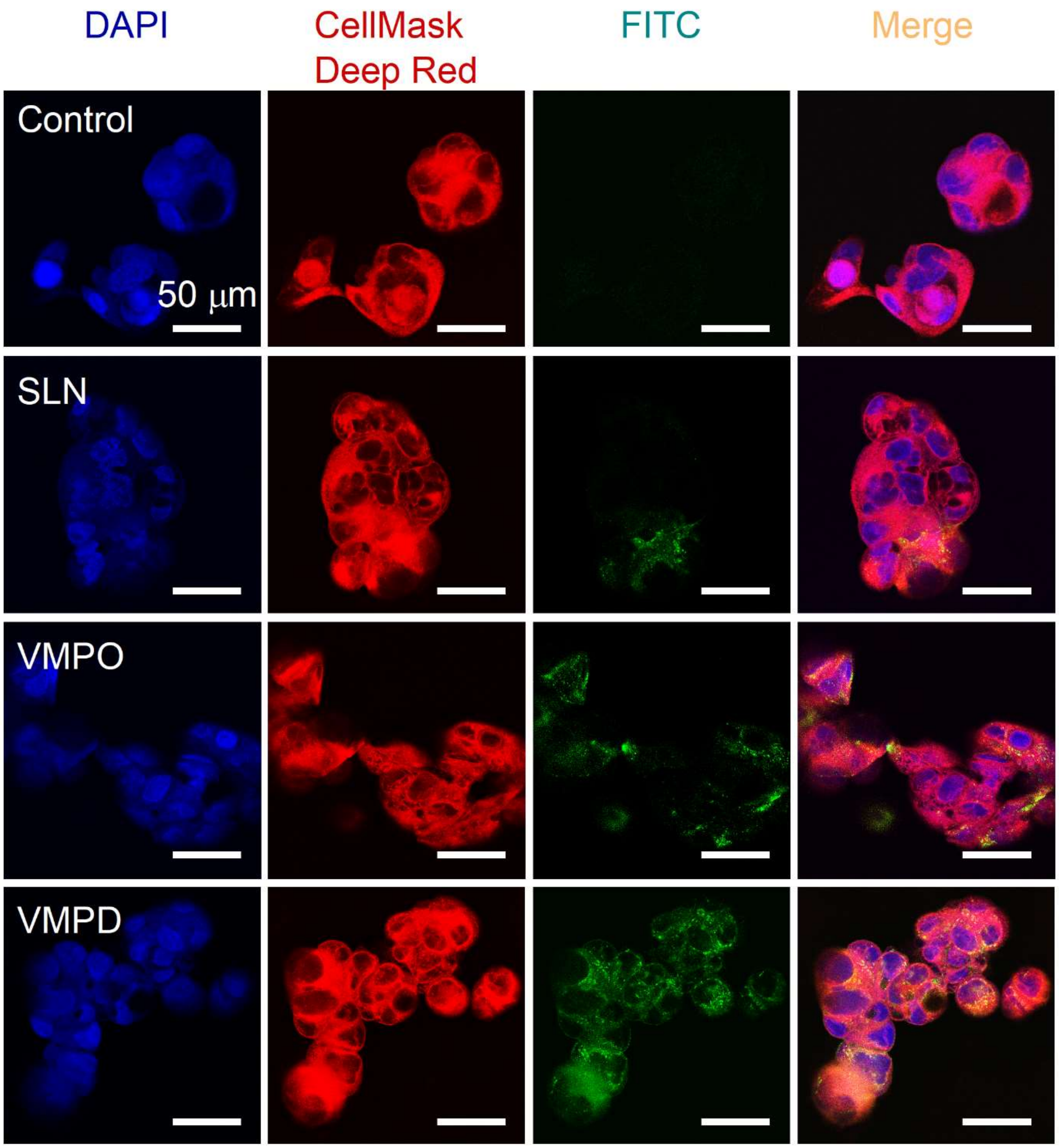

Figure S6. Laser scanning confocal microscope images revealing the cell uptake of FITC-labelled SLN, VMPO and VMPD (20 μg/mL) by Calu-3 cells after 3 h incubation.

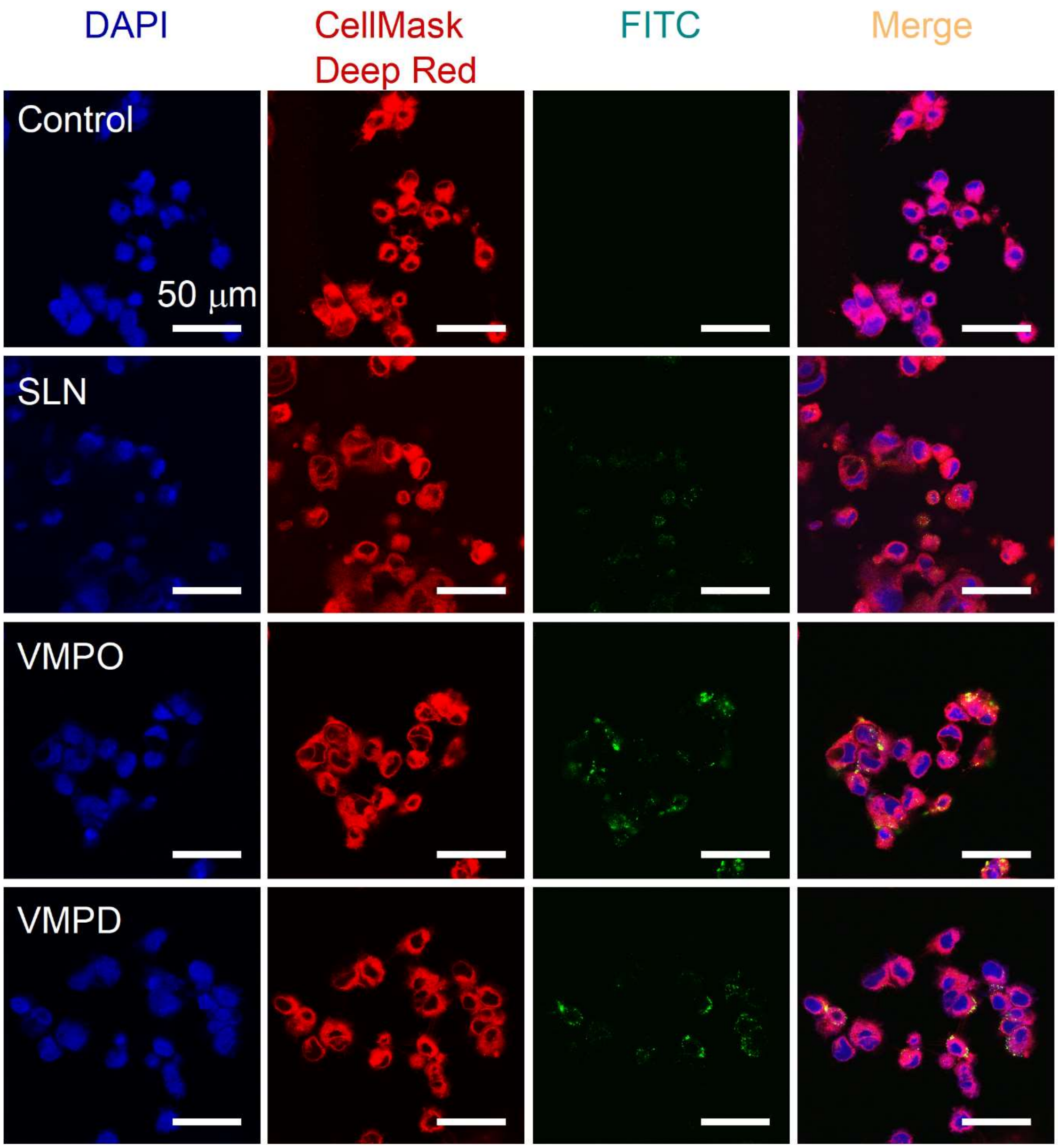

Figure S7. Laser scanning confocal microscope images revealing the cell uptake of FITC-labelled SLN, VMPO and VMPD (20 μg/mL) by Caco-2 cells after 3 h incubation.

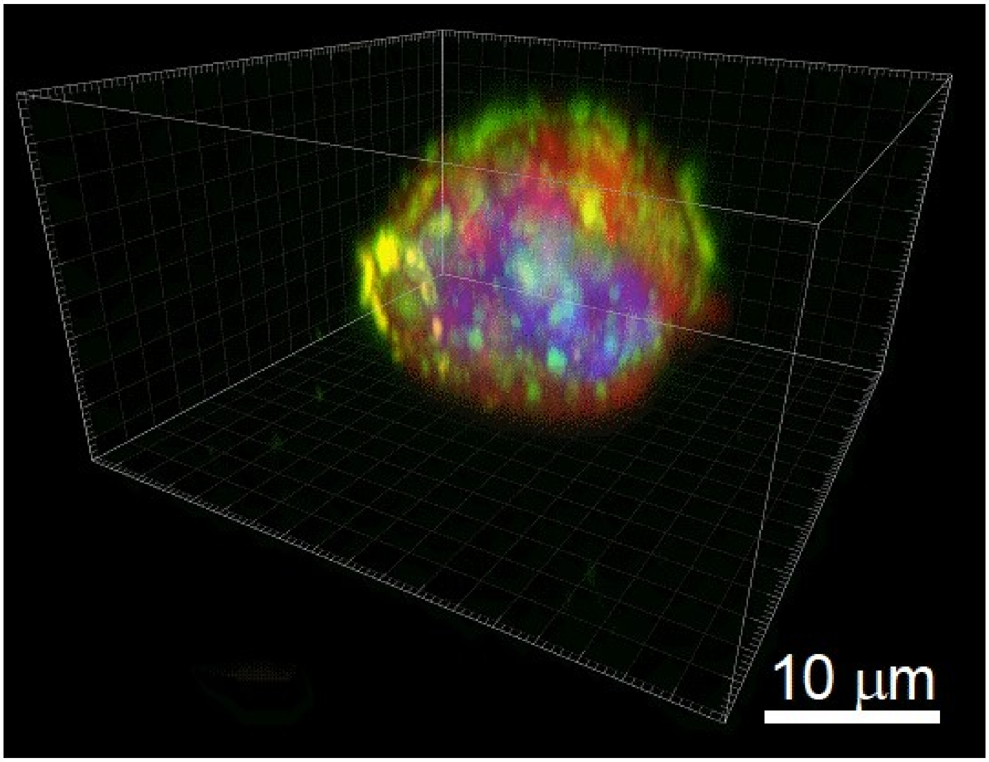

Figure S8. Confocal *z*-stack image of a Calu-3 cell treated with FITC-labelled VMPO (20 μg/mL) for 3 h.

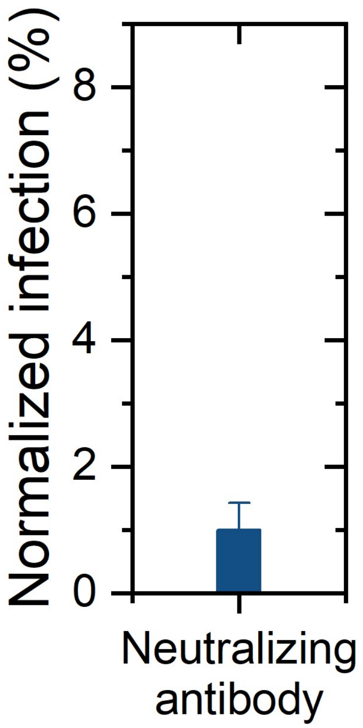

Figure S9. The effect of neutralizing antibody on blocking SARS-CoV-2 pseudoviral infection.

REFERENCES

(1) Zhang, P.; Du, C.; Huang, T.; Hu, S.; Bai, Y.; Li, C.; Feng, G.; Gao, Y.; Li, Z.; Wang, B.; et al. Surface adsorption-mediated ultrahigh efficient peptide encapsulation with a precise ratiometric control for type 1 and 2 diabetic therapy. *Small* **2022**, *18* (15), 2200449.

(2) Zhang, P.; Li, C.; Huang, T.; Bai, Y.; Quan, P.; Li, W.; Zhang, Z.; Zhang, F.; Liu, Z.; Wan, B.; et al. Inhibiting phase transfer of protein nanoparticles by surface camouflage–A versatile and efficient protein encapsulation strategy. *Nano Letters* **2021**, *21* (22), 9458-9467.

(3) Cerdá, S. L.; Fontana, F.; Wang, S.; Correia, A.; Molinaro, G.; Tello, R. P.; Hirvonen, J.; Celia, C.; Barreto, G.; Santos, H. A. Development of siRNA and budesonide dual-loaded hybrid lipid–polymer nanoparticles by microfluidics technology as a platform for dual drug delivery to macrophages: An in vitro mechanistic study. *Advanced Therapeutics* **2023**, *6* (8), 2300048.

(4) Lei, C.; Qian, K.; Li, T.; Zhang, S.; Fu, W.; Ding, M.; Hu, S. Neutralization of SARS-CoV-2 spike pseudotyped virus by recombinant ACE2-Ig. *Nature Communications* **2020**, *11* (1), 2070.
